# Modeling Percolation in Polymer Nanocomposites by Stochastic Microstructuring

**DOI:** 10.3390/ma8105334

**Published:** 2015-09-30

**Authors:** Matias Soto, Milton Esteva, Oscar Martínez-Romero, Jesús Baez, Alex Elías-Zúñiga

**Affiliations:** 1 Escuela de Ingeniería y Ciencias, Tecnologico de Monterrey, Campus Monterrey, Ave. Eugenio Garza Sada 2501, Monterrey, N.L. 64849, Mexico; matias.soto.castillo@gmail.com (M.S.); esteva@hotmail.com (M.E.); oscar.martinez@itesm.mx (O.M.-R.); 2 Centro de Estudios de Energía, Dpto. de Ing. Eléctrica, Tecnologico de Monterrey, Campus Monterrey, Ave. Eugenio Garza Sada 2501, Monterrey, N.L. 64849, Mexico; jesus.baez@itesm.mx

**Keywords:** carbon nanotubes, electrical properties, conductivity, percolation, Monte Carlo, nanocomposites, nodal voltage, simulations

## Abstract

A methodology was developed for the prediction of the electrical properties of carbon nanotube-polymer nanocomposites via Monte Carlo computational simulations. A two-dimensional microstructure that takes into account waviness, fiber length and diameter distributions is used as a representative volume element. Fiber interactions in the microstructure are identified and then modeled as an equivalent electrical circuit, assuming one-third metallic and two-thirds semiconductor nanotubes. Tunneling paths in the microstructure are also modeled as electrical resistors, and crossing fibers are accounted for by assuming a contact resistance associated with them. The equivalent resistor network is then converted into a set of linear equations using nodal voltage analysis, which is then solved by means of the Gauss–Jordan elimination method. Nodal voltages are obtained for the microstructure, from which the percolation probability, equivalent resistance and conductivity are calculated. Percolation probability curves and electrical conductivity values are compared to those found in the literature.

## 1. Introduction

The intrinsic electrical properties of carbon nanotubes (CNTs) have been found to be comparable to most metals in terms of conductivity and current density [1,2]. Depending on the nanotube chirality, its electrical conductivity can be classified as metallic or semiconducting [3,4,5,6]. Within a given group of single-walled carbon nanotubes (SWCNTs) grown inside the same furnace, researchers have found that approximately one-third are categorized as metallic, while two-thirds as semiconductors [3,5]. Metallic SWCNTs have a conductivity ranging from 10^4^ to 10^7^ S/m [6,7], while those classified as semiconductors have a conductivity of approximately 10 S/m [7].

Carbon nanotubes have been used as fillers to reinforce polymeric materials, and improvements in mechanical, thermal and electrical properties have been reported [2,3,8,9]. The properties of polymer nanocomposites (PNCs) can be engineered by manipulating the type and amount of fillers within the polymer matrix. Furthermore, these parameters can be used to tailor the electrical properties of the nanocomposite [8,9]. Having a model that incorporates the abovementioned parameters is essential to quantify their impact in the final electrical properties of the PNC. Previous theoretical and computational work has been reported in the open literature, which focuses on the prediction of the percolation threshold and electrical conductivity of PNCs [10,11,12,13,14,15,16,17,18,19].

### 1.1. Percolation Behavior

A polymer nanocomposite is composed of carbon nanotubes embedded in a polymer matrix. The fillers can be well dispersed, bundled and dispersed, or completely agglomerated. Either the bundles or the singular CNTs can be aligned in a particular direction or misaligned. When a specific electrical current source is supplied to a polymer nanocomposite with carbon nanotubes as fillers, there will be a defined transition from an insulator to a conductor in the electrical conductivity. This transition occurs because a conductive path has been established within the carbon nanotube network. The term percolation refers to this onset of transition to a network where connectivity suddenly appears [15,20].

For composites with conductive fillers dispersed in an insulating matrix, the percolation of a polymer nanocomposite may be directly related to the concentration of fillers inside the matrix. As this concentration is increased, the nanotubes are able to interact with each other to form the conductive network necessary for the transport of electrons across the material, and the concentration at which this occurs is known as the percolation threshold [3]. The contact percolation limit, which is associated with the establishment of a nanotube conductive path from one boundary to another in a polymer nanocomposite, has been estimated between 0.12 and 4.5 wt. % for different polymer matrices (polyimide, epoxy, *etc.*). In addition to the effect on electrical conduction due to an increase in the filler concentration within the polymer matrix, it is important to mention that percolation is also relevant when describing the mechanical properties of PNCs, so that a change in the behavior is observed around the percolation threshold, according to [21,22].

### 1.2. Electrical Conductivity

In general, the conductivity of a medium is defined as the inverse of resistivity, which is an intrinsic property of that same medium. Materials considered as conductors have a low electrical resistivity, while those considered as insulators have high values of resistivity. Semiconductors are between conductors and insulators [23].

In the case of polymer nanocomposites, the electrical conductivity is dependent on the percolated network because the nanotubes are embedded inside the polymer matrix. Unless percolation is established, the transport of electrons from one boundary to another will hardly occur. After percolation has been obtained within the PNC, the conductivity continues to be dependent on the concentration of the nanotubes [3]. It is well-known that not only the concentration of nanotubes has an effect on the conductivity of a PNC, but also their dispersion and agglomeration because this relates to the formation of contact conductive networks [12,14].

The electron hopping or quantum tunneling phenomenon also affects the electrical conductivity of a PNC [16], such that in cases where the filler concentration does not allow for the direct contact among nanotubes, if the separation distance between them is small enough, electrons have been detected to be transported within the polymer material [16,24]. The quantum tunneling barrier or resistance depends on the properties of the polymer, the distance between the two segments showing tunneling behavior, and the voltage applied at the junction. This characteristic behavior in polymer nanocomposites enhances its electrical conductivity [16].

In this work, we present a methodology capable of simulating the electrical properties of a polymer nanocomposite with carbon nanotube as fillers. The simulation can calculate values of percolation probability and electrical conductivity. In addition, the results of the nodal voltage analysis can be graphed to create a map of the voltage distribution within the electrical network.

Our work shares several characteristics with previous works and also proposes some novel methods when modeling the microstructure and finding the conductive nanotube network within it. Although many recent works have a three-dimensional microstructure, this work along with that of Li *et al.* [13] employs a two-dimensional approach. Nanotube waviness has been previously used in the model of Dalmas *et al.* [11] and Li *et al.* [13], and also incorporated into our model. The concept of electron tunneling has already been added to the models reported in [12,16,18], while contact resistance is part of the models previously reported in [11,16,17]; both of which were also incorporated into our model. The works of [12,14,17,18] use a special algorithm to find the conductive path in the microstructure; instead of using a similar approach, in our work we make use of linear algebra methodology in order to discriminate when a nanotube is connected to the network that conducts electric current. Aside from the aforementioned characteristics that our work shares with previous models, this methodology makes use of length and diameter distributions when generating the microstructure. Additionally, the waviness of a nanotube depends on its length based on a linear relation. Finally, the intrinsic conductivity of the nanotubes in our model is assigned according to a distribution found experimentally in [2].

## 2. Procedure

A methodology was established to simulate the electrical behavior of a polymer nanocomposite with carbon nanotubes as fillers. Firstly, the generation of the geometrical parameters of the PNC model is explained. Secondly, the network interactions within the microstructure are discussed, which includes crossings and tunneling behavior. This is followed by the conversion of the microstructure and its interactions to an equivalent electrical circuit. Finally, the equivalent electrical circuit is then used in the nodal analysis.

As we will see, the use of the nodal voltage analysis method allows us to determine percolation of the fiber network as well as its electrical conductivity. This nodal voltage analysis is coupled with the use of linear algebra methods needed to obtain the voltage at each node in all the nanotubes in the network.

### 2.1. Generating and Analyzing the Microstructure

**Figure 1:**
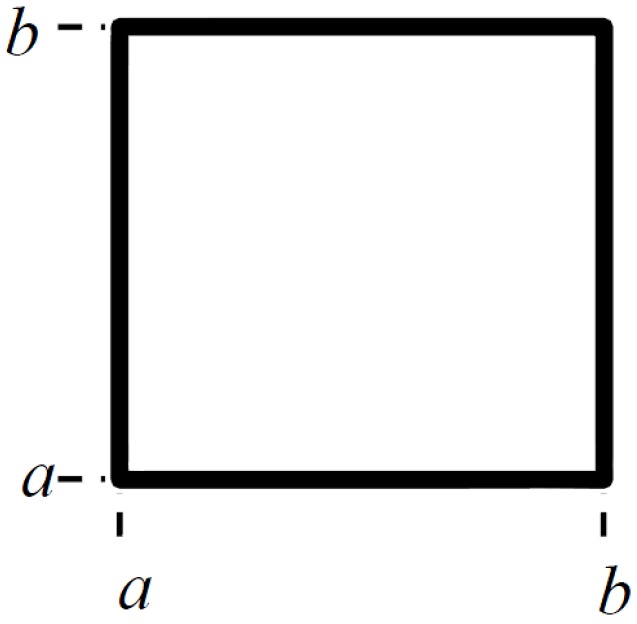
Top view of Representative Volume Element (RVE).

A network of fibers was generated within a representative volume element (RVE). The area of the RVE was determined to be a square (for simplicity) of side length (b–a) (see Figure 1). The thickness, *t*, of the RVE was chosen as the maximum nanotube diameter. When using a specific distribution for the nanotube diameter, the maximum diameter to a 95% confidence level was used.

#### 2.1.1. Generating the Geometry

Each fiber was generated as a series of straight segments with initial and final nodes. A pseudorandom number generator is used for this purpose. The generator uses a uniform distribution of numbers in the open interval (*a*, *b*). In this case, the generator was first used to obtain two numbers, the *X* and *Y* coordinates of a single point, which gives the location of the first node of the segment. With this basic coordinate pair, this node is saved as an Object, using hierarchical object-oriented programming in Matlab. The Object, which can be simply called Node, may also save other information about the node; this proved to be advantageous when administering large data structures in the computer algorithm.

The second node is created at a distance *l* from the first node. The segment distance, *l*, depends on the fiber distance and the number of segments, such that,

(1) L=kl

where k is the number of segments and L is the sum of all the individual segment lengths in a fiber. The proportion of the total length of the fiber to the RVE side length (*b*–*a*) will be referred to as the parameter *g* in some portions of this article.

**Figure 2:**
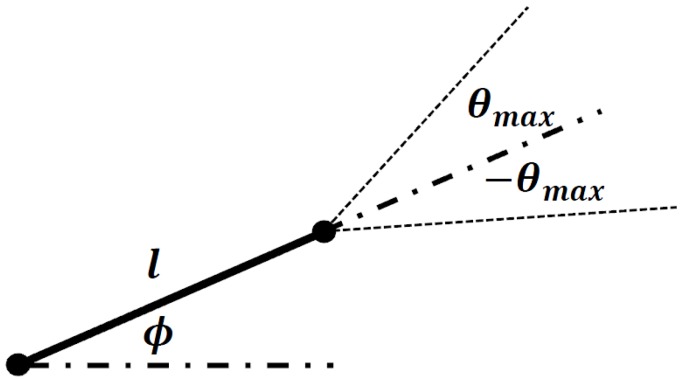
Angle of deviation of the second segment with respect to the previous one.

The same pseudorandom number generator is used to determine the orientation of the second node with respect to the first one. In this case, the open interval covers all 360 degrees such that the second node can be randomly-oriented in any direction with respect to the first node, as shown in Figure 2. The angle of orientation, ϕ, is then used to find the *X*–*Y* coordinates of the second node according to the following equations:

(2) $$X_{\text{i}+1}=X_{\text{i}}+l\cos\phi$$

(3) $$Y_{\text{i}+1}=Y_{\text{i}}+l\sin\phi$$

**Figure 3:**
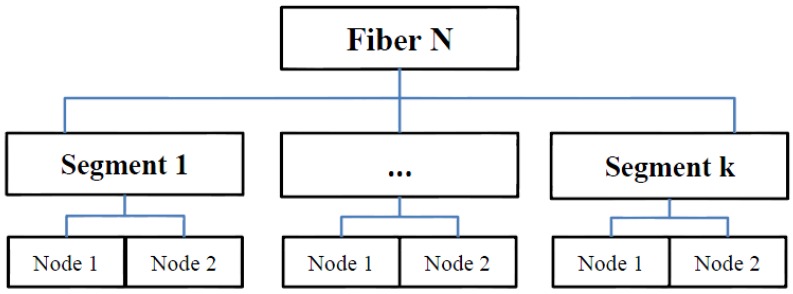
Object hierarchy of the computer algorithm.

**Figure 4:**
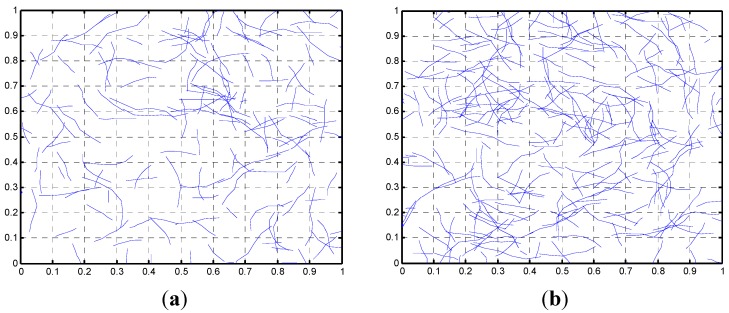
Network of fibers with lengths following a Weibull distribution (**a**) *c* = 0.01 and (**b**) *c* = 0.02.

The second node is also saved as a Node Object. As part of the hierarchy, an Object called Segment is created, which stores the two Node objects. The next segment makes use of the second node (node i+1 in Figure 2), from which a third node is generated. The angle of orientation of this next segment is limited by the boundaries that define the maximum deviation of the next segment with respect to the previous angular deviation, as shown in Figure 3. The same procedure is followed for the generation of the remaining segments for each fiber. As mentioned before, each Segment object is composed of two Node objects; in turn a Fiber object can be created, which is composed of *k* Segment objects. The complete hierarchy of the Objects is shown in Figure 4.

Multiple Fiber objects can be generated to form a network. The volume fraction of the fillers is calculated by summing the volumes of all the individual fibers and dividing by the volume of the RVE.

(4) $$c=\frac{V_{all\;fibers}}{V_{RVE}}$$

The volume of each fiber is given by the following equation:

(5) $$V_{f}=\overset{k}{\underset{i=1}{\sum }}\pi \frac{d^{2}}{4}l_{i}$$

where d is the diameter of the fiber, and the volume of the RVE is given by the following equation:

(6) $$V_{RVE}=t{\left(b-a\right)}^{2}$$

Additionally, the aspect ratio (AR) of a fiber is defined as the ratio of its total length to its diameter, which is assumed to be constant.

When generating the geometry, the length, diameter, and maximum angle of deviation (waviness, in essence), among other parameters, can be defined in our methodology. Following the work done by Spanos and Esteva [2], the lengths of the fibers and the diameters follow a distribution, and the maximum angle of deviation can be computed from a linear relationship. The fiber length, *x*, follows a Weibull distribution of the form:

(7) $$x=\beta {\left(-ln\left(1-u\right)\right)}^{\frac{1}{\alpha }}$$

where *α* and *β* are the shape and scale parameters found to be 2.4 and 161.74 by analyzing the data of Wang *et al.* [25], and where the variable u is a uniformly distributed random number between 0 and 1. The mean value (length) for the aforementioned parameters is 143 nm. The microstructures shown in Figure 4 were obtained for 0.01 and 0.02 volume fractions with parameters *α* = 2.4 and *β* = 161.74. Notice from Figure 4 how the fiber length varies.

The diameters of the fibers are defined according to a log-normal distribution from a fitting study by Spanos and Esteva [2]. The log-normal probability density function (PDF) determined in [3] follows a distribution given as:

(8) $$f\left(x\right)=\frac{1}{x\sigma \sqrt{2\pi }}e^{-\frac{{\left(lnx-\mu \right)}^{2}}{2\sigma^{2}}}$$

with a mean μ=0.02847 and standard deviation σ=0.3363 of the variable’s natural logarithm. The mean of the diameter can be found by using the following relation,

(9) $$Mean_{dia}=e^{\mu +\sigma^{2}/2}$$

**Figure 5:**
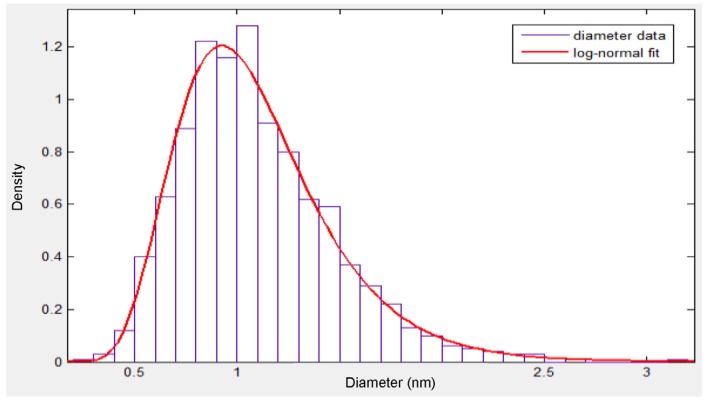
Log-normal distribution of the nanotube diameters.

Using the above values of μ and σ, the mean diameter is found to be 1.089 nm (nanometers) with a standard deviation of 0.3767, which falls within the range of single-walled carbon nanotubes (SWCNTs). The histogram of such a distribution is shown in Figure 5 for 1000 values generated randomly using the function lognrnd in Matlab and along a log-normal fit. The curve fit was generated using the dfittool command. Notice from Figure 5, that there is good agreement between the randomly-generated log-normal data and the log-normal fit.

The maximum angle of deviation (see Figure 2) must also be specified. As proposed by [2], a linear relationship exists between the maximum angle of deviation of a segment, and the length of the nanotube. The relation is given by,

(10) y=0.375x−7.5

where *x* is the nanotube length in nm and *y* is the maximum angle of deviation in sexagesimal degrees.

#### 2.1.2. Network Interactions

**Figure 6:**
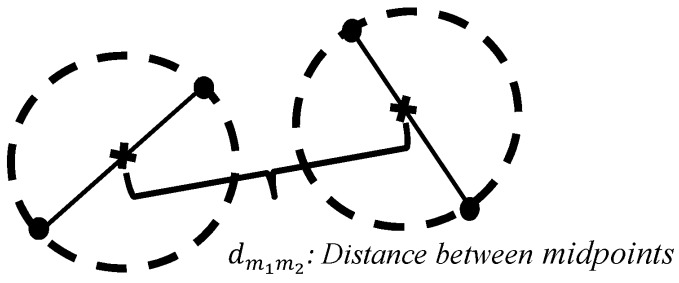
Calculation of distance between midpoints for two segments.

A differentiation control is performed on the Segment objects to find the different network interactions by selecting a fiber and comparing its segments with all the other segments of the other fibers. First, the radius of the two segments being compared is calculated as half the distance between their initial and final nodes. Second, the coordinates of the midpoints of both segments are located. Third, the distance between the two midpoints is calculated (see Figure 6); this distance is then compared with the sum of the radii of both segments, as in the following equation:

(11) $$D=r_{1}+r_{2}$$

If the distance between midpoints ($d_{m_{1}m_{2}}$) is less than or equal to the parameter *D*, then the segments are considered to be “close enough”.

**Figure 7:**
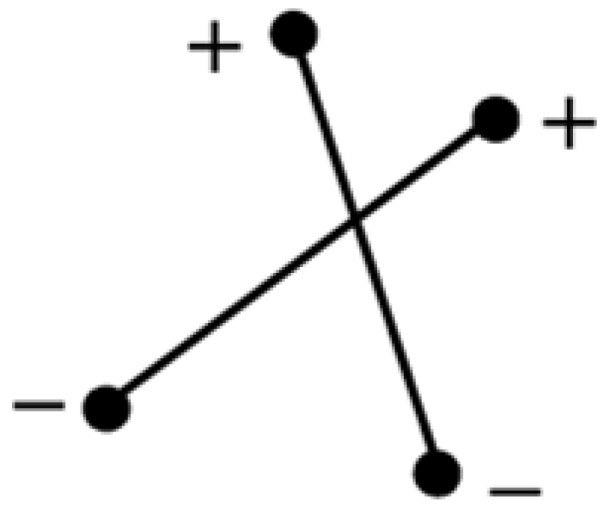
Sign differences between node-segment distances.

If two segments are considered to be “close enough”, then a second comparison is used to find if the two segments are crossed. To do that, first we calculate all the distances between the nodes of both segments using the following equation:

(12) $$d=\frac{\left|y-mx-b\right|}{\sqrt{m^{2}+1}}$$

where *m* and *b* are the slope and *y*-intercept of the straight line segment, and *x* and *y* are the coordinates of the location of the node. By removing the absolute value of Equation (12), the sign of the number provides additional information that can be used in the algorithm. If both nodes with x and y coordinates are on the same side of any given line described by the parameters *m* and *b* of Equation (12), then their distances will have equal signs; for nodes on opposite sides of such straight line, their distances will have opposite signs. For two segments to be crossed, the distances between the nodes of segment one and straight segment two will have opposite signs; and the distances between the nodes of segment two and straight segment one will also have opposite signs, as shown in Figure 7.

For the two segments that are crossed, the computer algorithm separates them by the crossing point into two new segments. These Segment objects will be composed of one of its original Node objects and the crossing point as the other Node, will be part of the same Fiber object as before, and inherit all the properties of the original “mother” segment.

**Figure 8:**
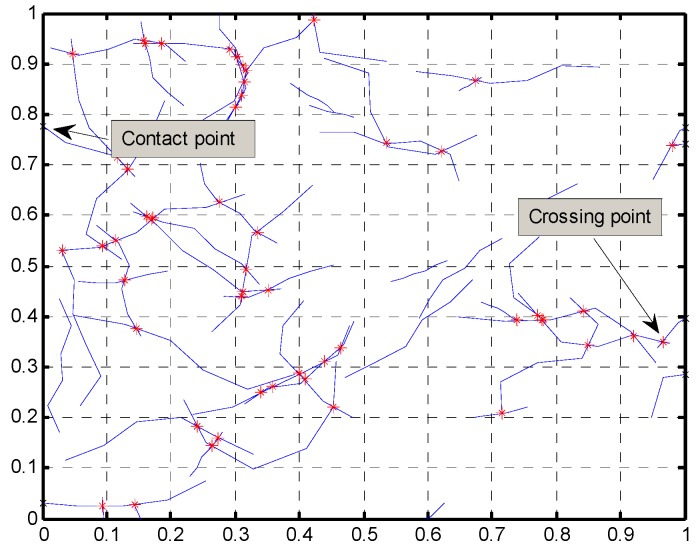
Network of fibers with crossing points between their segments.

Contact with left and right boundaries were also accounted for in the model by simply comparing the *x*-coordinate of the segment nodes to the *x*-coordinate values of the RVE’s left and right boundaries. Figure 8 below shows boundary contact points marked with a crossmark and crossing points between segments marked with an asterisk.

**Figure 9:**
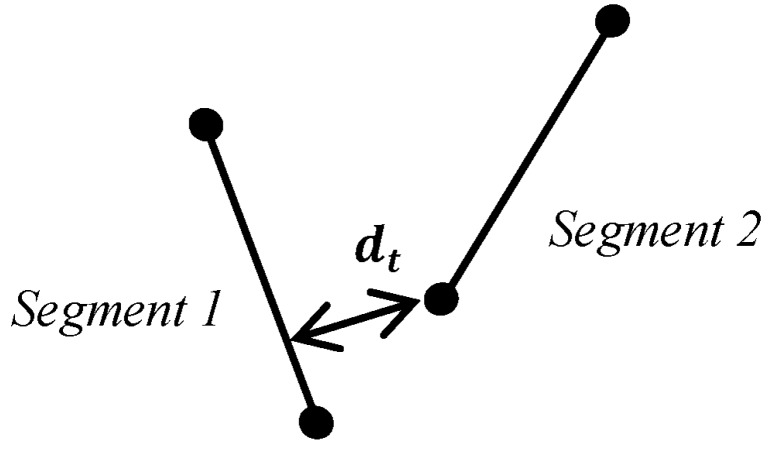
Shortest distance between an end node and another segment.

Tunneling segments were included into the model by comparing if distances between segments were less than a predefined threshold. For two non-parallel straight segments in a two-dimensional plane, the shortest distance between them will always include an end point of one of these segments. This is the basic principle underlying the process of finding tunneling paths. If two segments have been found to be “close enough” by the previous differentiator, then the tunneling path testing begins. For this test, the four distances between the four end nodes and the corresponding opposite segments are calculated, as shown in Figure 9.

**Figure 10:**
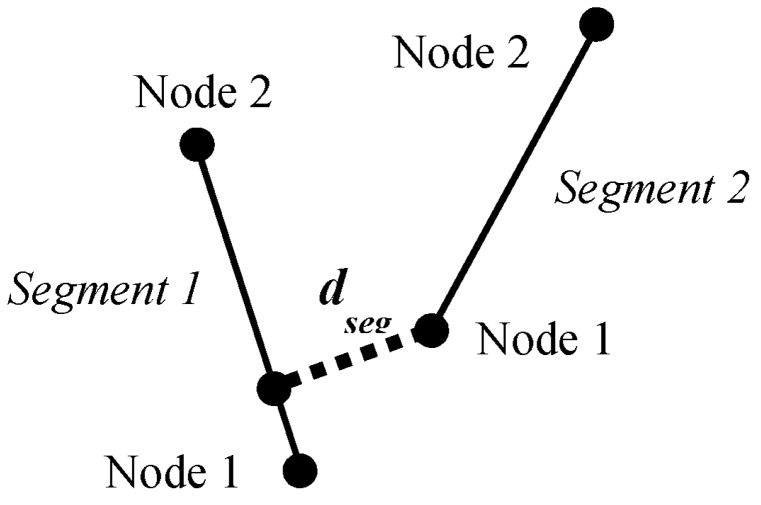
Tunneling path between a node and a segment.

**Figure 11:**
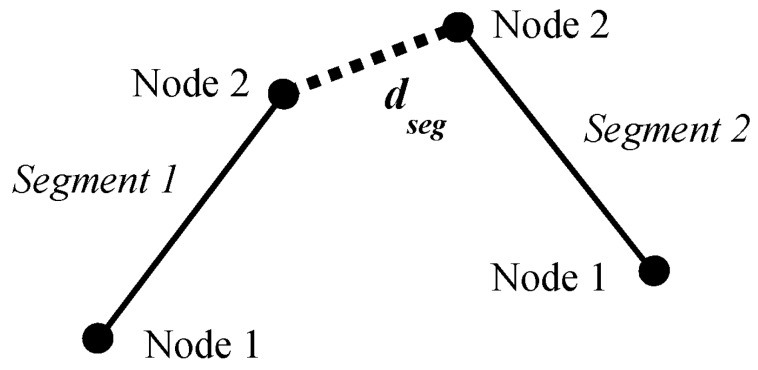
Tunneling path between two nodes.

When any of these fours distances are less than or equal to the defined distance, *d*, then a tunneling path is created. This path becomes a new Segment object with initial and final nodes as any other segment in the microstructure. There are two possible cases for tunneling paths: one with a path between a node and a segment (Figure 10) and another one with a path between two nodes (Figure 11).

The distance threshold for the establishment of a tunneling path for different polymer materials was taken from Sun *et al.* [16], and shown in Table 1. According to Sun *et al.* [16], the tunneling distance is the point at which an increase in length beyond a specific threshold gives a conduction equal to zero. Below the distance threshold, tunneling conduction is a significant contributor to the overall electrical resistivity of the network and can be considered as part of the equivalent electrical circuit.

**Table 1:** Minimum distances for tunneling paths. All values taken from Sun *et al.* [16].

Matrix Material	Distance (nm)
Polyethylene (PE)	2.00
Polyimide (PI)	2.50
Polyvinyl Alcohol (PVA)	2.27

### 2.2. Solution via Node-Voltage Analysis

Node-voltage analysis consists of deriving equations for each node in the circuit (except for a reference node) by applying Kirchhoff’s current law. This is the principle underlying the methodology used to calculate the voltage at each node in the microstructure. In this methodology, each element in the microstructure is treated as a resistor, and then the resistor network is analyzed at each node.

#### 2.2.1. Converting Fiber Network into Set of Equations

According to several authors [11,12,17,26,27], a polymer nanocomposite may be modeled as a resistor network for simulation purposes. In this network, each nanotube is modeled as a resistor. The polymer matrix may be modeled as an insulating material by assigning it an electrical resistance much higher than the nanotubes. In our case, each segment is modeled as an electrical resistor, depending on its intrinsic resistivity (*ρ*), diameter, and length. If such resistivity is assumed to be constant along the material, as well as having a material with a constant cross-sectional area (assuming a circular solid rod as a model for the SWCNT), then the electrical resistance may be calculated by the following relation:

(13) $$R=\frac{\rho L}{A_{n}}$$

where *L* is the length of the medium; and *A*_n_ is the cross-sectional area of the circular solid rod modeling the CNT; and *ρ* is the electrical resistivity.

Tunneling resistance paths are included in the resistor network by using the following relation from Xu *et al.* [18]:

(14) $$R_{tunnel}=\frac{h^{2}d}{A_{t}e^{2}\sqrt{2m\lambda }}exp\left(\frac{4\pi d}{h}\sqrt{2m\lambda }\right)$$

where *e* is the quantum of electricity; *h* is Planck’s constant; m is the electron mass; *A*_t_ is the cross sectional area of the tunnel formed between CNTs; *d* is the distance of the tunnel; and *λ* is the average barrier height, which depends on the material of the polymer matrix. A list of average barrier heights taken from Sun *et al.* [16] and Xu *et al.* [18] is summarized in the following Table 2.

**Table 2:** Average barrier height of various polymer materials.

Matrix Material	Barrier Height (eV)
Polyethylene (PE)	4.43 [16]
Polyimide (PI)	4.56 [16]
Polyvinyl Alcohol (PVA)	2.58 [16]
Epoxy	0.5–2.5 [18]

In order to account for the increase in electrical resistance when two segments are in contact or in close proximity, the electrical resistance for such segments was calculated according to Equation (13), and increased 200% from its original value. This must be included in the model because the crossed segments provide a higher electrical resistance to the flow of electrons. The increase percent was taken from a molecular dynamics study by Buldum *et al.* [28].

**Figure 12:**
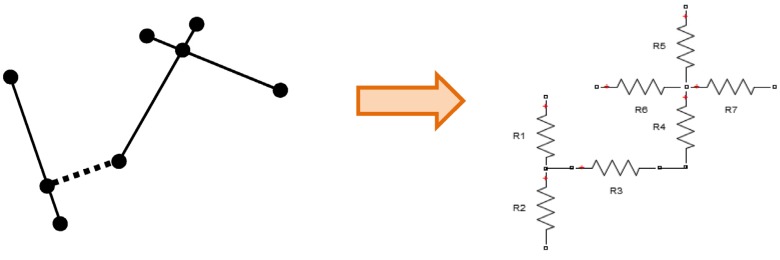
Schematic of modeling a fiber network as a resistor network.

The process of modeling the nanotube segments and tunneling paths as resistors is represented schematically in Figure 12, in which *R*1 and *R*2 are calculated using Equation (13), *R*3 is a tunneling path and is calculated using Equation (14), and *R*4 through *R*7 have a resistance according to Equation (13) but increased 200% because of contact resistance.

After each element in the network has been assigned a value of resistance, the following matrix equation taken from [27], is formed:

(15) $$\left[G_{N}\right]\lfloor {\bar{V}}_{N}\rfloor =\lfloor {\bar{I}}_{N}\rfloor$$

where the term $\left\lfloor {\bar{V}}_{N}\right\rfloor$ is a column vector containing the nodal voltages; $\left\lfloor {\bar{I}}_{N}\right\rfloor$ is also a column vector of all the current sources into the circuit; and *G*_N_ is the conductance matrix. For the case of a circuit with only electrical resistors and sources, this matrix is also symmetric [29].

**Figure 13:**
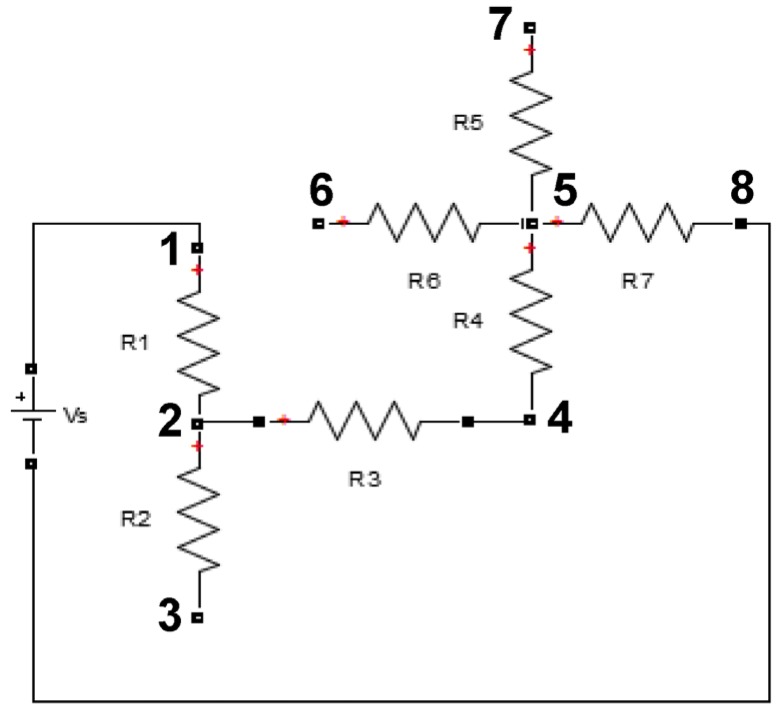
Example case of a resistor network.

**Figure 14:**
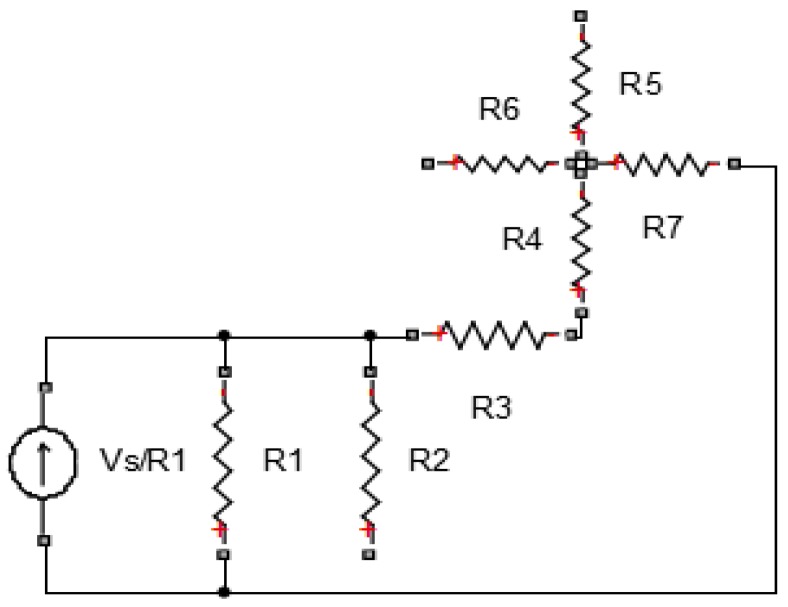
Transformed example resistor network.

To illustrate the process of deriving the set of equations, the resistor network shown in Figure 13 will be analyzed. The current sources vector is formed using the current sources connected at each node. In the example below, the current entering node 2 is obtained by transforming the circuit from having a voltage as a source to a resistance in parallel with a current source, as shown in Figure 14. After this transformation, the current source is equivalent to *V*_s_/*R*_1_.

**Figure 15:**
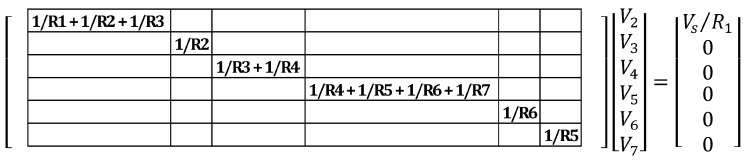
Diagonal elements of conductance matrix for example case.

In our case, the left-hand side boundary is connected to a current source, while the right-hand side boundary is connected to the ground (voltage equal to zero). The current source column vector is formed by the algebraic sum of the source currents entering each node, while using the same nodal numbering to arrange the vector in the correct order, as seen in Figure 15. If a node is connected to the left-hand side boundary via one or more resistors, Ohm’s Law is used to add the value of each source current entering the node.

After the current sources vector is formed, the conductance matrix can be populated. Nodal analysis states that the diagonal elements of the conductance matrix are formed by adding the conductance of the resistors connected to each node [29] (conductance is defined as the inverse of resistance). For the network of Figure 13 and Figure 14, the diagonal elements of the conductance matrix are shown below in Figure 15.

**Figure 16:**
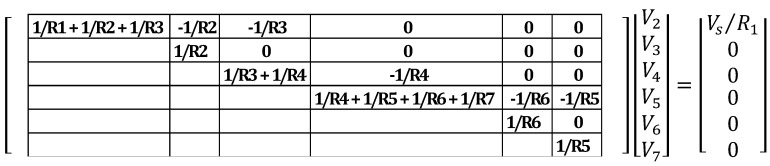
Top non-diagonal elements of the conductance matrix

**Figure 17:**
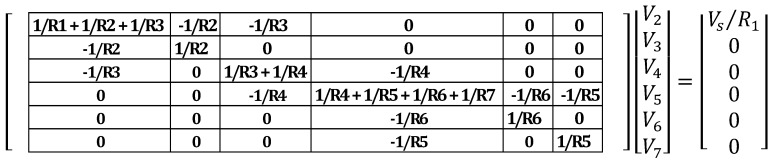
Complete conductance matrix of example case.

Once the diagonal elements of the conductance matrix have been filled, the non-diagonal elements must also be identified and added to the matrix. According to nodal analysis, the non-diagonal elements of the j-th row of the conductance matrix are zero, except those of the columns corresponding to nodes directly connected to the j node by a resistor, which value is the conductance of the connected resistor, with a negative sign [29]. Updating the conductance matrix, the diagonal and top non-diagonal elements of the matrix are shown in Figure 16. The bottom non-diagonal elements can simply be added by noting that the matrix is symmetric about the diagonal (see Figure 17).

#### 2.2.2. Boundary Conditions

The RVE shown in Figure 1 is a three-dimensional space with an electrical potential between the left- and right-hand side boundaries. In the work by Dalmas *et al.* [11], this potential difference was set to 200V, while all the other boundaries are considered as electrically insulated (zero electrical current flow through them). The same potential difference was used in this work while solving the system of Equation (15). The left-hand side boundary acts as the voltage source and the right-hand side boundary can be considered as connected to the ground.

#### 2.2.3. Solving System of Equations

Once the set of equations has been formed and the boundary conditions defined, as shown in the previous sections, then the system can be solved for the nodal voltages. The method used in this work for solving the system of linear equations is Gauss-Jordan elimination. The main advantage of this method is that it can discriminate between the independent and non-independent equations in a linear system. When generating a conductance matrix for a random network of fibers, it is highly probable that some of the fibers will not be connected to a “main” network. The “main” network refers to the group of fibers that are connected to each other and where at least one is connected to the voltage source.

The nodes of the fibers that are not connected to the “main” network or to the source boundary will not have an independent equation in the set. In previous works, other authors resorted to using an algorithm to determine a conduction path that would eliminate from the set of equations the fibers that are not connected to this conduction path before generating the conductance matrix. In this way, the conductance matrix is guaranteed to be nonsingular. Instead of resorting to such algorithm for path finding, a pure mathematical solution, Gauss-Jordan method, can be used to solve the system of equations. The Gauss-Jordan method generates a matrix of zeroes and ones only. The non-independent rows can be identified because they will composed of only zeroes after being reduced by the Gauss–Jordan elimination method. These rows of zeroes are sent to the bottom of the system of equations, after which, the newly-modified conductance matrix is multiplied by the current source vector to find the nodal voltages of Equation (15).

**Figure 18:**
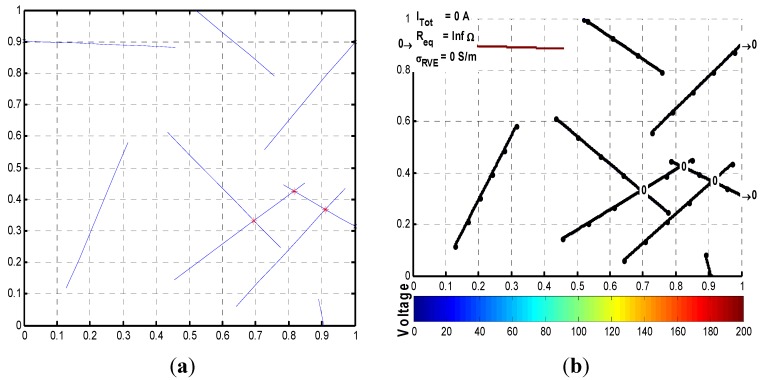
(**a**) Microstructure of straight nanotubes, *c* = 0.01, AR = 130; and (**b**) corresponding nodal voltage diagram.

**Figure 19:**
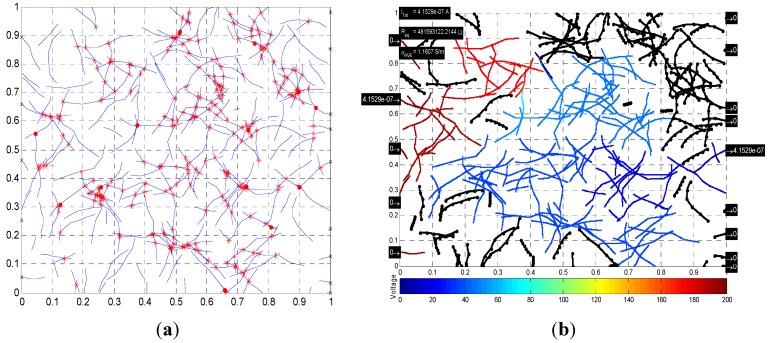
(**a**) Microstructure with length/diameter distributions, *c* = 0.015; and (**b**) corresponding nodal voltage diagram.

The information obtained from the nodal voltage analysis can be used to visualize the electrical properties of the RVE. The diagrams in Figure 18 and Figure 19 show the voltage distribution: a *colorbar* is used to show how the voltage varies in the microstructure; those fibers that are not connected to the voltage source or the “main” network are colored in black; and, the insulated polymer matrix is simply shown in white. Additionally, electrical currents incoming on left-hand side boundary and electrical currents outgoing on right-hand side boundary are shown (Figure 18b), where the units are in Amperes. The sum of incoming currents must be equal to the sum of outgoing currents, which are also equal to the total current in the circuit. The equivalent resistance of the RVE is also shown on the diagram, along with the corresponding electrical conductivity. This information summarizes the electrical properties of the RVE.

A microstructure of straight nanotubes is shown first in Figure 18. The nanotubes not connected to the voltage source directly or through another nanotube are shown in black, which means that the voltage distribution across them is zero. The nanotube that is connected to the voltage source but not connected to the ground is as in an open circuit configuration, and the voltage across it is 200 V. As expected, the total current and RVE conductivity of Figure 18 are both zero.

Figure 19 shows a microstructure with length and diameter distributions, generated using the parameters described in Section 2.1.1. Here, the incoming and outgoing electrical currents are shown on the diagram. Incoming and outgoing electrical currents of those nanotubes that are connected to boundaries but in open circuit configuration are zero. In addition, the sum of incoming currents equals the sum of those outgoing, and both sums are equal to the total current in the circuit, which is shown on the top left corner of Figure 19b along the equivalent resistance and conductivity of the RVE. Nodal voltages of each crossing are not shown numerically because of the amount of crossings in the diagram, but the color code indicates how the voltage is being distributed in the RVE.

## 3. Results and Discussion

### 3.1. Percolation Behavior

**Figure 20:**
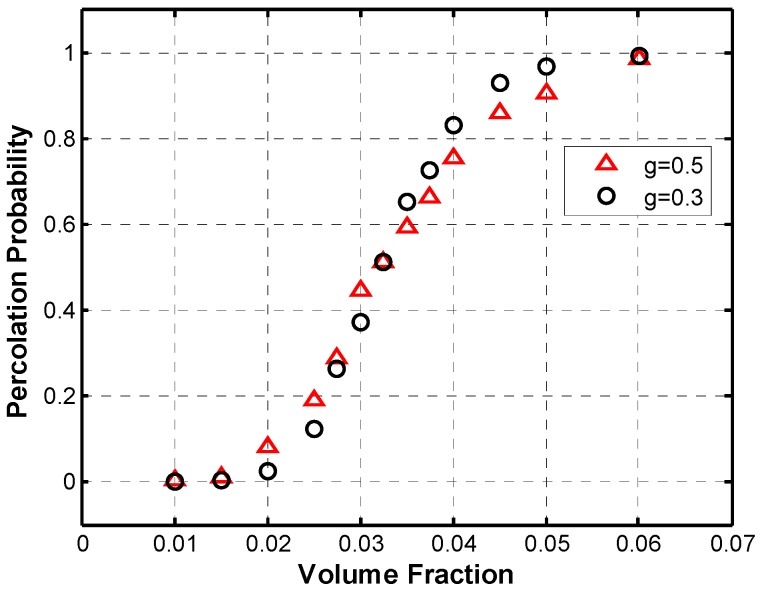
Percolation probability curves intersect at the percolation threshold, for AR = 140.

**Figure 21:**
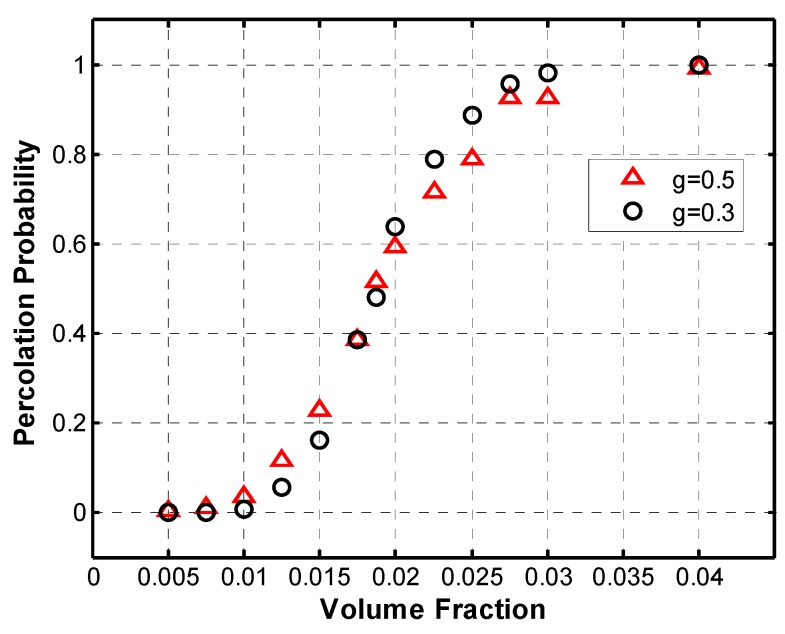
Percolation probability curves intersect at percolation threshold, AR = 240.

**Figure 22:**
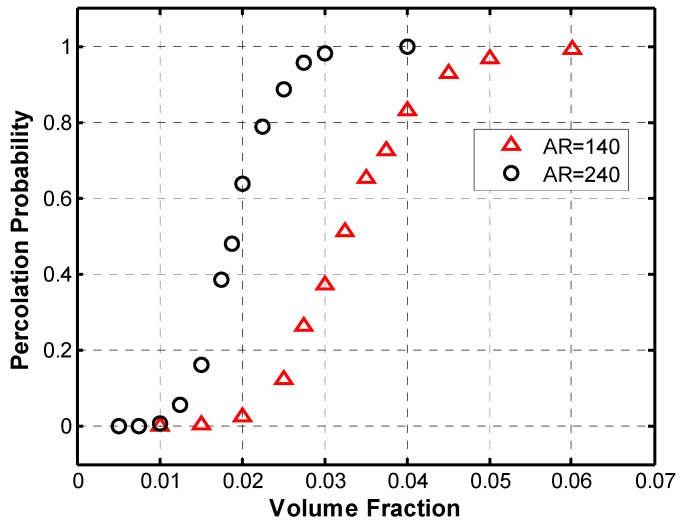
Comparison of percolation probability curves with different aspect ratios, *g* = 0.3.

**Figure 23:**
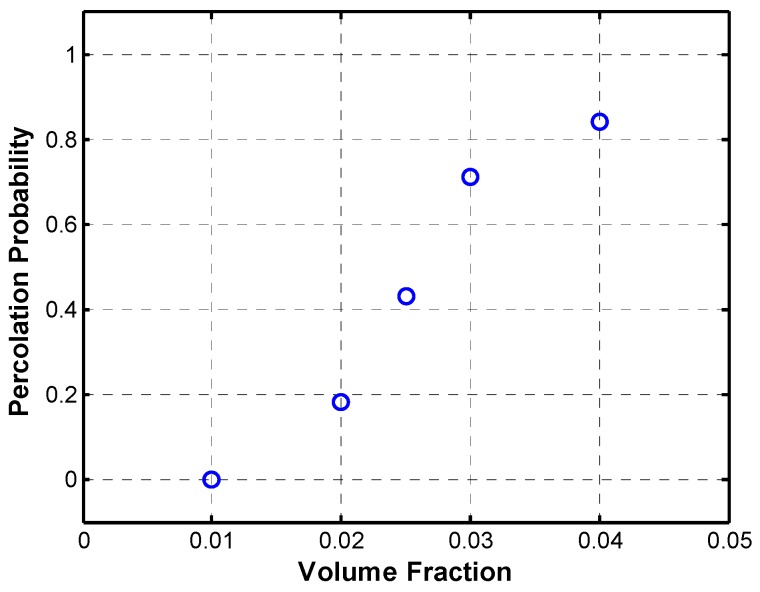
Percolation probability with length and diameter statistical distributions.

First, the percolation behavior of the simulated microstructures was analyzed and compared to similar work in the literature. In order to do that, Monte Carlo simulations were performed for different set of parameters. The percolation probability was calculated as the ratio of successfully percolated microstructures over the number of simulations performed. Thus each data point in the graphs below (Figure 20, Figure 21, Figure 22 and Figure 23) represents several Monte Carlo simulations for a single set of parameters.

The fillers in the polymer nanocomposite, carbon nanotubes in this case, are said to percolate when they form a network which is able to conduct electricity across the polymer matrix. A sudden increase in percolation probability is expected from theory, but because a limited volume is being simulated, a smooth increase in percolation probability is seen instead (Figure 20), which is also observed in the work of Flandin *et al.* [20].

The two curves in Figure 20 show the percolation behavior for the simulation of microstructures with constant length and diameter distributions for all nanotubes with an aspect ratio of 140 and a maximum angle of deviation of 45°. The only difference between the curves is in the length of the fibers, (a) 500 nm and (b) 300 nm. Figure 20 shows the typical percolation behavior described in literature [3,15].

The crossing point between the curves gives the percolation threshold for the given parameters, which occurs at 3.25 vol. % and at a percolation probability of approximately 0.5. This is consistent with Zeng *et al.* [19], which makes use of a percolation probability of 0.5 as an indicator of the electrical percolation threshold in their Monte Carlo simulations. It can also be noted that the curve with *g* = 0.03 has a steeper slope at the critical area where the percolation probability rapidly increases. A sudden increase in percolation probability is expected from theory, thus it can be observed that decreasing the parameter g has the effect of generating a percolation curve, which tends to approach the theoretical sudden increase.

Keeping all the other parameters constant, the aspect ratio was increased from 140 to 240, and a range of volume fractions were simulated for *g* = 0.5 and *g* = 0.3. From Figure 21, it can be seen that the curves intersect at two different points, at 1.75 vol. % and again at approximately 1.9 vol. %. The corresponding percolation probability is 0.386 and about 0.55, respectively, close to the 0.5 percolation probability value, as expected from [19].

Figure 22 compares two percolation curves with the same fiber length parameter, *g*, but with different aspect ratios. Increasing the aspect ratio (AR) shifts the curve to the left, from which it can be observed that the increase in the aspect ratio decreases the volume fraction at which the percolation threshold occurs, which is consistent with previous literature [13]. Moreover, from the figure below, it can be noted that for the same volume fraction, a higher percolation probability can be obtained for the case of aspect ratio of 240, thus a higher electrical conductivity would in turn be achieved.

The values for percolation threshold obtained are comparable to experimental values from the literature. Table 3 shows the comparison with data taken from Potschke *et al.* [30]. Additionally, we may compare these results to the pioneering work of Flandin *et al.* [20]. In their work, they simulated the percolation behavior of random three-dimensional microstructures made up entirely of straight fibers with an aspect ratio of about 14, and found a percolation threshold of 4.45% ± 0.05%. Although the models are different in several aspects and the results in percolation threshold comparable, the disagreement could be mainly attributed to the difference in aspect ratio of the fillers.

**Table 3:** Comparison of percolation threshold results with values from other authors.

Author	Aspect Ratio	CNT Type	Matrix Type	Percolation Threshold
This work	140–240	SWCNTs	PE (thermoplastic)	1.75–3.25 vol. %
Potschke *et al.* [30]	100–667	MWCNTs	PC (thermoplastic)	1.47–2.94 vol. %

Additional to the results in percolation behavior shown above, an improvement upon previous simulations was made to portray the microstructure more accurately with length and diameter statistical distributions and length-dependence of the maximum angle of deviation. Monte Carlo simulations were performed to calculate the percolation probability for the microstructure with the aforementioned distributions, and the results are shown in Figure 23. For these simulations, the log-normal diameter distribution used had a mean diameter of 2.18 nm; and the Weibull fiber length distribution had a mean value of 0.29 µm. Fibers were generated with five segments per fiber in an RVE of 1 µm side length and a thickness equal to the maximum diameter to a confidence level of 95%, which was calculated to be 2.85 nm. The percolation threshold can be estimated to be between 2.5 and 3 vol. %, which is comparable to the experimental results by Potschke *et al.* [30].

### 3.2. Tunneling Behavior

**Figure 24:**
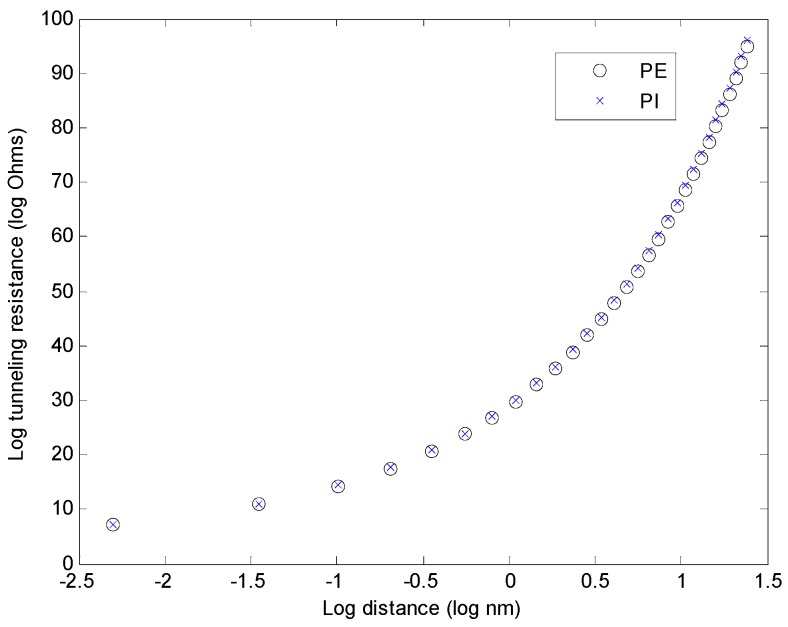
Tunneling resistance as a function of distance between nanotubes.

The tunneling cut-off distances and the average barrier height values taken from [16] and the equation to calculate tunneling resistance, taken from [18] and shown as Equation (14), were used to calculate the tunneling resistance as a function of the distance between segments for two polymer matrices, PE and PI. From the figure below (Figure 24), it can be seen that when the distance between two nanotubes decreases, the tunneling electrical resistance decreases too. Such resistance seems to increase rapidly at a distance near 2 nm, which agrees with previous work [16].

**Figure 25:**
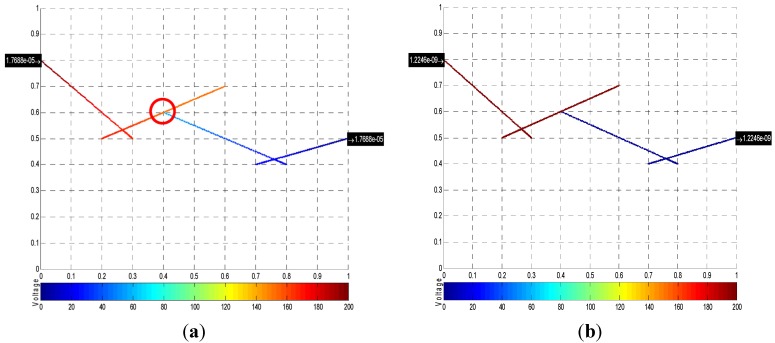
Tunneling path of (**a**) 0.45 nm and (**b**) 0.89 nm.

The distance between two nanotube segments can affect the voltage distribution in the microstructure. A simple comparison it is shown to illustrate this point: a tunneling path is formed between two segments (formed inside the red circle in Figure 25a); for this particular case, the tunneling path is 0.45 nm long and has a resistance of 1.0 × 10^7^ Ω. If the separation between the segments is increased to 0.89 nm, the resistance increases to 3.2 × 10^11^ Ω. As expected, the voltage drop across the tunneling path also changes dramatically: from 0.20 V in the first case to 190 V in the second case. Additionally, increasing the separation between the segments decreases the electrical current from 1.77 to 1.23 A, which in turn would affect the conductivity of the RVE.

At a tunneling path length of 1.92 nm, the resistance is already 3.0 × 10^21^ Ω and the voltage drop is approximately 200 V. Keeping in mind that the total voltage drop across the RVE is 200 V, the flow of electrons would be nearly impossible for tunneling paths longer than 2 nm, which agrees with the cut-off distances of Table 1.

#### 3.3. Electrical Conductivity

The conductivity of the material was also calculated from Monte Carlo simulations, which included tunneling behavior, contact resistance, and microstructure with length and diameter distributions and length-dependent maximum angle of deviation. The mean diameter of the log-normal distribution was set at 2.18 nm, the mean fiber length of the Weibull distribution was set at 0.29 µm, with five segments per fiber. A side length of 1 µm and thickness of 2.85 nm was set for the RVE. The conductivity distribution was set with metallic intrinsic conductivity of 10^5^ S/m and semi-conductor intrinsic conductivity of 10 S/m, potential difference of 200 V, tunneling cut-off distance of 2 nm, and a barrier height of 4.43 eV.

**Figure 26:**
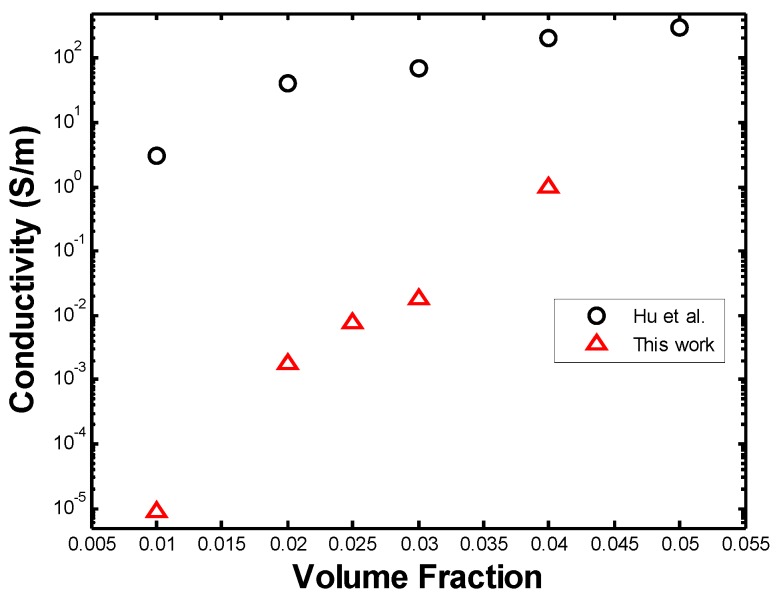
Comparison of PNC conductivity to the work by Hu *et al.* [12].

**Figure 27:**
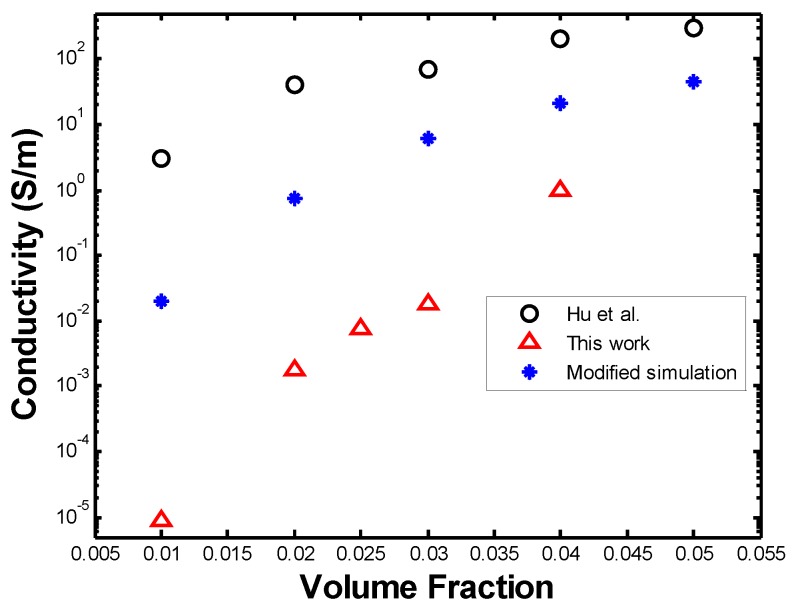
Comparison of [12] with modified simulation.

Each data point plotted in Figure 26, Figure 27, and Figure 29 is the mean conductivity value of the several Monte Carlo simulations performed for each set of parameters. These conductivities were compared to the values obtained from simulations by Hu *et al.* [12] (see Figure 26). Hu *et al.* performed 3-D Monte Carlo simulations of straight CNTs with an AR = 100, length of 5 µm, diameter 50 nm, and a constant distribution of intrinsic CNT conductivity of 10^4^ S/m. The values of conductivity obtained by Hu *et al.* are clearly larger than the ones obtained in this work, although both curves follow a similar trend. This is as expected, namely because [12] uses a constant CNT intrinsic conductivity of 10^4^ S/m, while this work assumes that one-third of the CNTs are metallic and with a conductivity of 10^5^ S/m and two-thirds behave as electrical semi-conductors with an intrinsic conductivity of 10 S/m. In addition, Hu *et al.* [12] does not take into account the contact resistance generated when CNTs come into contact, which increases the electrical resistance of CNTs and of the RVE overall.

To obtain a better comparison to [12], a range of volume fractions between 0.01 and 0.05 was simulated with a constant fiber length of 500 nm and aspect ratio of 100, and using a constant intrinsic conductivity of 10^4^ S/m for all the nanotubes generated in the microstructure and ignoring contact resistance. The RVE side length was set at 1 µm and the thickness, in this case, was set equal to the fiber diameter, which is 5 nm.

Figure 27 shows the original values of conductivity along with the ones from the modified simulation, being compared with those obtained by [12]. It can be seen that, by defining a constant intrinsic conductivity for all the nanotubes and ignoring contact resistance, a higher PNC conductivity can be obtained, which tends towards the values obtained by [12]. As seen from the figure above, a discrepancy of at least one order of magnitude in the conductivity is seen between both studies. The RVE in our work is essentially two-dimensional, however, a thickness must be defined in order to calculate its conductivity. Such thickness was defined as the diameter of the fibers, namely 5 nm, while Hu *et al.*, who generates a three-dimensional microstructure in their simulations, uses the dimensions of their three-dimensional RVE to calculate its conductivity. If the thickness of the RVE is increased in our work, a higher value of conductivity is obtained and similar values to those of Hu *et al.* [12] may be obtained. However, this solution to the discrepancy is arbitrary and this problem must be further studied.

**Figure 28:**
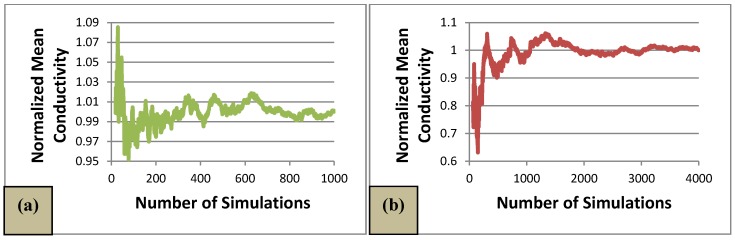

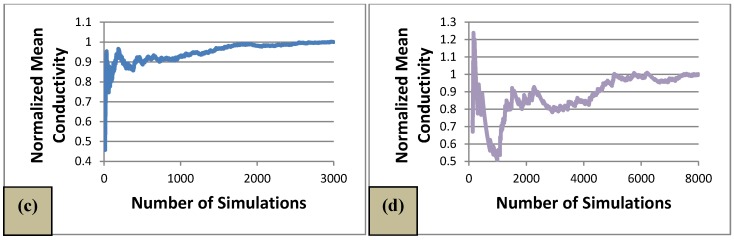
Normalized mean conductivity (**a**) *c* = 0.05; (**b**) *c* = 0.04; (**c**) *c* = 0.03; and (**d**) *c* = 0.02.

The number of simulations needed for convergence was analyzed; for this purpose, the mean conductivity after each simulation was normalized with respect to the final value. For a volume fraction of 0.05, after about 700 Monte Carlo simulations, the mean conductivity does not vary more than 1% with respect to the final value (see Figure 28a). A volume fraction of 0.04 requires more simulations to find a converging mean conductivity. Figure 28b shows that after about 2500 simulations, the mean value does not vary more than 1% with respect to the final mean conductivity. Similarly, volume fraction of 0.03 converges at about 3300 simulations, and 0.02 volume fraction at about 7400 simulations.

When performing Monte Carlo simulations for a volume fraction of low percolation probability (less than 0.01), only the few percolated cases exhibit conductivity, thus the mean value becomes somewhat volatile and close to zero. The number of simulations needed for convergence was too great for the purpose of this work. The value presented as the mean conductivity is only a rough approximation of the estimated converged value.

**Figure 29:**
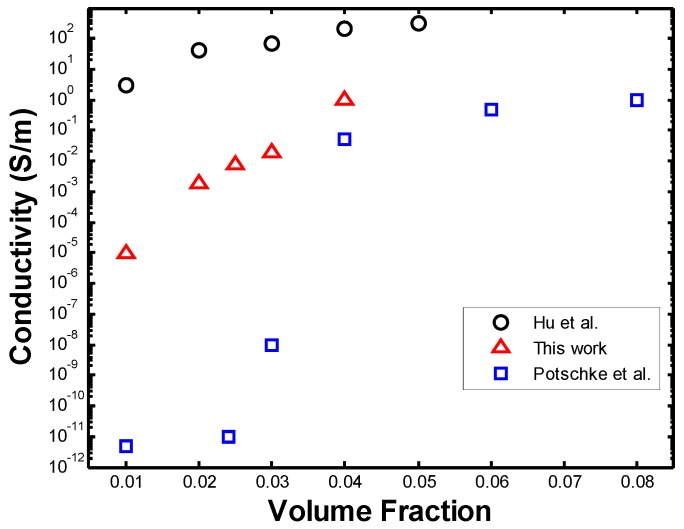
Comparison of conductivities to simulation and experimental data.

The conductivities were also compared to experimental values [30]. In their work, Potschke *et al.* measured the resistivity of PNCs with MWCNTs with diameters between 10 and 15 nm and length between 1 and 10 µm in a polycarbonate (PC) matrix. It can be seen in Figure 29 that the experimental data obtained from Potschke *et al.* is in closer agreement with the values obtained in this work than those from the simulation by [12]. The discrepancy in the values ranging from 0.01 and 0.03 volume fractions between our work and those obtained experimentally by [30] can be attributed to a difference in the exact location of the percolation threshold. For the work in [30], a sudden drop in conductivity occurs below 0.04 volume fraction, while our work shows a steady decrease until 0.02 volume fraction. Ultimately, this discrepancy could also be attributed to the difference in polymer matrix.

## 4. Concluding Remarks

This work contributes by developing a two-dimensional simulation of a microstructure with Weibull distribution for the nanotube length, a log-normal distribution of the nanotube diameter, as well as a relation used for the calculation of the maximum angle of deviation of a segment with respect to the previous segment, taken by the work done by [2]. The main purpose of incorporating these improvements to the simulation was to be able to generate a microstructure more representative of a carbon nanotubes network inside a polymer matrix. Additionally, this work includes tunneling behavior to the two-dimensional simulation, which has been included in previous works. The simulation is completed by incorporating an intrinsic nanotube conductivity distribution when calculating electrical resistance to each segment in the microstructure, and by adding contact resistance to the crossed segments. Finally, the simulation does not require the use of a path search algorithm to find the network of nanotubes that expand from one boundary to the other. Instead, a purely mathematical solution is employed to solving the set of equations generated from the nodal analysis, in which the non-independent equations are identified and removed from the set of equations by using the Gauss-Jordan method.

The computational methodology identified and developed can be used as an aid in the study and development of polymer nanocomposites. It is capable of generating a microstructure to a specified CNT volume fraction or specific number of CNTs and with the capability of adjusting several parameters such as fiber length, diameter, waviness, number of segments per fiber, and even including distributions for fiber length and diameter. The methodology can calculate relevant parameters in the study of the electrical properties of PNCs, such as percolation probability, equivalent electrical resistance, total electrical current flow in the RVE, nodal voltages for the entire microstructure, and conductivity of the RVE. The relevant parameters were put together in a diagram that summarizes the electrical properties of a particular microstructure; some examples of these diagrams are shown in Figure 18 and Figure 19.
